# Concurrent Primary Aldosteronism and Renal Artery Stenosis: An Overlooked Condition Inducing Resistant Hypertension

**DOI:** 10.3389/fcvm.2022.818872

**Published:** 2022-03-03

**Authors:** Lin Zhao, Jinhong Xue, Yi Zhou, Xueqi Dong, Fang Luo, Xiongjing Jiang, Xinping Du, Xianliang Zhou, Xu Meng

**Affiliations:** ^1^Department of Cardiology, Fuwai Hospital, National Center for Cardiovascular Disease, Chinese Academy of Medical Sciences and Peking Union Medical College, Beijing, China; ^2^Department of Cardiology, The Fifth Central Hospital of Tianjin, Tianjin, China

**Keywords:** primary aldosteronism, renal artery stenosis, renin, resistant hypertension, diagnosis

## Abstract

To explore the clinical features of coexisting primary aldosteronism (PA) and renal artery stenosis (RAS), we retrospectively analyzed records from 71 patients with PA with RAS and a control group of 121 patients with PA without RAS. Aldosterone-to-renin concentration ratio tests and computerized tomography (CT) scanning of the adrenal and renal arteries were routinely conducted to screen for PA and RAS. Color Doppler flow and/or magnetic resonance imaging were used as substitute testing of patients for whom CT was contraindicated. Standard percutaneous renal arteriography (PTRA) was considered for patients with RAS exceeding 70% based on non-invasive tests and for those without PTRA contraindications. The patients with PA with RAS were further divided into severe (RAS>70%) and moderate (50% < RAS <70%) RAS groups. The prevalence of RAS among PA patients was 6.9% (71/1,033), including 3.2% (33/1,033) with severe RAS. Compared with the PA without RAS group, the severe RAS group showed higher levels of systolic blood pressure (SBP) (171.82 ± 18.24 vs. 154.11 ± 18.96 mmHg; *P* < 0.001) and diastolic BP(DBP) (110.76 ± 15.90 vs. 91.73 ± 12.85 mmHg; *P* < 0.001) and prevalence of resistant hypertension (RH) (90.9 vs. 66.9%; *P* = 0.008), whereas the moderate RAS group merely showed higher DBP (98.63 ± 14.90 vs. 91.73 ± 12.85 mmHg; *P* = 0.006). The direct renin concentrations (DRCs) (5.37 ± 3.94 vs. 3.71 ± 2.10 μU/mL; *P* < 0.001) and false-negative rate (33.8 vs. 3.3%; *P* < 0.01) of PA screening tests were significantly higher in the PA with RAS group than in the control group, but only in severe RAS group, in subgroup analysis. Among patients who underwent successful treatment for severe RAS, mean DRC decreased from 11.22 ± 9.10 to 3.24 ± 2.69 μIU/mL (*P* < 0.001). Overall, the prevalence of RH decreased from 81.7 to 2.8% (*P* < 0.001) when both PA and RAS were treated with standard methods. PA with concurrent severe RAS is a condition that induces RH. PA can be easily missed in patients with coexisting RAS. RAS patients with RH after successful revascularization for RAS should be evaluated for coexisting PA.

## Introduction

Primary aldosteronism (PA) is the most common cause of secondary hypertension (HTN) and accounts for 10–20% of all-cause secondary HTN cases (1, 2). PA is characterized by excessive and autonomous secretion of aldosterone that is independent of renin and angiotensin II (Ang-II). Excessive aldosterone promotes sodium and water reabsorption, which causes volume expansion and the development of HTN (3). Endothelial dysfunction, vascular remodeling (4, 5), sympathetic nervous system (SNS) activation (6), and baroreflex dysfunction (7) induced by aldosterone excess may be additional mechanisms by which HTN develops in patients with PA. However, the detection rate of PA in the community with newly diagnosed HTN is only about 4% (8), and the detection rate for PA in patients under the care of HTN specialists was 10.7% (9).

Renal artery stenosis (RAS) remains a significant cause of secondary resistant HTN [RH, RH is defined as uncontrolled BP that remains above goal with appropriate lifestyle measures and the use of 3 or more antihypertensive agents, one of which should be a diuretic (10)]. The prevalence of RAS is 2% in all HTN cases, 5.8% in young adults with secondary HTN, and 6.8% of patients > 65 years old (11). RAS has been widely studied as the prototype of angiotensin-dependent HTN. Goldblatt et al. (12) demonstrated that the regulation of the BP was far more complex than merely the regulation of blood volume by the kidney. His demonstration that unilateral RAS causes HTN without significant volume expansion spurred the search for other mechanisms of BP homeostasis including the renin-angiotensin-aldosterone system (RAAS) (13). RAS induces excessive activation of the RAAS because the low blood flow and pressure to affected kidney stimulate the release of renin, which further increase the synthesis of Ang-II and aldosterone (14, 15). In spite the effects of aldosterone, increased Ang II in RAS also recruits additional pressor mechanisms, including sympathetic adrenergic pathways, vasoconstriction and vascular remodeling, inflammatory and fibrogenic mechanisms (16, 17).

The aldosterone-to-renin concentration ratio (ARR) is recognized as the initial screening test for PA followed by confirmatory tests and subtype diagnostic tests (18, 19). However, although considered the most dependable screening test for PA, false-positive and false-negative ARR findings are not rare because the RAAS is affected by various factors, including the presence of RAS (20–22). In patients with concurrent PA and RAS, the balance within the RAAS is disrupted. The increased renin in RAS makes the ARR an inaccurate screening tool for PA. As a result, the diagnosis and treatment of PA may be delayed. The coexistence of PA and RAS is a rare occurrence in clinical practice, and only a few sporadic cases have been reported (23–25). Research on the clinical manifestations and diagnostic testing among patients with both PA and RAS remains limited. This retrospective study summarized findings from 71 typical patients with concurrent PA and RAS, and compared them with findings from a control group of 121 patients with PA without RAS, to reveal the clinical and laboratory features among PA and RAS patients.

## Methods

### Patients and Clinical Data

Patients with PA and RAS who were hospitalized consecutively at Fuwai Hospital from January 1, 2008 to December 30, 2019 formed the “PA with RAS” group. Inclusion criteria were as follows: (I) met the diagnostic standards for PA in accordance with the Endocrine Society clinical practice guidelines (18), and (II) RAS was > 50%. Among patients diagnosed with PA after revascularization for RAS, those presenting with recurrent or aggravated HTN after 6 months after interventional surgeries were excluded because their PA might have represented a new onset after the revascularization treatment of RAS. RAS in the range of 50–70% and > 70% was defined as moderate and severe, respectively (26). In accordance with this definition, patients with both PA and RAS were divided into severe and moderate RAS groups to permit subgroup analysis of clinical and laboratory characteristics. To get similar follow-up data to that of PA with RAS group, the consecutive patients with PA and no evidence of RAS who were admitted to the hospital from January 1, 2017 to December 31, 2017 were selected for the “PA without RAS” group.

This study complied with the Declaration of Helsinki (World Medical Assembly) and its amendments and was approved by the Ethics Committee of Fuwai Hospital. Informed consent from the subjects was not required because this was a retrospective study.

### Diagnosis of PA and RAS

All patients in our center suspected to have secondary HTN underwent screening tests for common etiologies, including both PA and RAS. ARR tests and computerized tomography (CT) scans of the adrenal and renal arteries were routinely conducted to screen for PA and RAS. Color Doppler flow and/or magnetic resonance imaging were used as substitute tests for patients for whom CT was contraindicated. Standard percutaneous renal arteriography was considered for patients with RAS that exceeded 70% based on non-invasive tests and those without arteriography contraindications.

Patients underwent screening and confirmatory tests for PA according to the Endocrine Society clinical practice guidelines (18). The ARR was used as the case-detection test for PA and was calculated by dividing the plasma aldosterone concentration (PAC) by either the plasma renin activity (PRA) (before 2016) or the direct renin concentration (DRC) (since 2016). A subsequent confirmatory test (see below) was conducted when: the orthostatic ARR was > 30 ng dL^−1^/ng mL^−1^ h^−1^ (using PRA) or > 3.7 ng dL^−1^/μIU mL^−1^ (using DRC), which was defined as a positive result, and PAC was higher than 15 ng/dL. A probable positive result was defined as an ARR ranging from 20 to 30 ng dL^−1^/ng mL^−1^ h^−1^ (using PRA) or 2.4 to 3.7 ng dL^−1^/μIU mL^−1^ (using DRC). A negative ARR referred to < 20 ng dL^−1^/ng mL^−1^ h^−1^ (using PRA) or < 2.4 ng dL^−1^/μIU mL^−1^ (using DRC). Diagnosis of PA was confirmed if the PAC was reduced to < 30% of the baseline level in the captopril challenge test or remained > 10 ng/dL after saline injection (27). Because PA is defined as excessive, uncontrolled, autonomous aldosterone secretion, acute captopril or saline injections do not result in a significant decrease in plasma aldosterone levels (28). PRA (ng/mL/h) was converted to DRC (μIU/mL) using a conversion factor of 8.2 (18) only when renin profiling was involved in the statistical analysis. In order to reduce confounding factors from existing medications, all patients halted their antihypertensive drugs that might affect the RAAS for 4 weeks prior to screening and confirmatory testing. Alpha-blockers and calcium channel blockers, which have little impact on the RAAS, were used to control blood pressure (BP) in all patients (29). Patients with hypokalemia received slow-release potassium chloride to maintain serum potassium concentrations in the range of 3.5–5.5 mmol/L. Normal dietary sodium intake for at least 5 days was also required in all patients.

Adrenal CT scanning was routinely conducted for the initial subtype classification of PA in all patients. Adrenal lesions were classified as thickened or nodular according to the imaging characteristics. Adrenal thickening was diagnosed when the adrenal limb was ≥ 1 cm or was thicker than the ipsilateral diaphragmatic crura (30). The adrenal glands are located above the kidneys on both sides, and they can each be anatomically divided into a body and two limbs. The origin of the posterior portion of the diaphragm is known as the diaphragmatic crura. The right diaphragmatic crus begins at the 1st−3rd lumbar vertebral bodies and intervertebral fibrocartilage, and the left diaphragmatic crus begins at the 1st−2nd lumbar vertebral bodies and intervertebral fibrocartilage.

Adrenal vein sampling (AVS) is performed when adrenalectomy is being considered (31). In the present study, AVS was carried out by an experienced physician in accordance with an established protocol and without adrenocorticotropic hormone stimulation (32). A ratio of the cortisol concentration in the adrenal vein to the average value in the superior and inferior vena cava >2 indicated successful AVS. Unilateral PA was defined as a cortisol-corrected aldosterone level from one side that was two-times higher than the other side (19).

### Definitions and Data Collection

Two trained researchers retrospectively extracted data describing patient demographics, medical histories, and laboratory and imaging findings. Potential duplicate records with matching birth year, sex, and clinical features were eliminated. Low renin status was defined as a DRC < 15 μIU/mL or PRA < 0.65 ng/mL/h (33). The disease duration was calculated from the onset of HTN and the diagnosis of PA. A false-negative result from the screening test was defined as follows: the result was negative in the first test independent of whether there were potential confounding factors, and PA was finally diagnosed during another hospital admission. The false-negative rate (FNR) was the ratio of the number of false-negative ARR patients to all patients being tested. The estimated glomerular filtration rate was calculated using the Chronic Kidney Disease Epidemiology Collaboration Equation (2009) with creatinine detected by an enzymatic method (34). Body mass index was calculated with the following formula: body mass index (kg/m^2^) = weight (kg)/height^2^ (m^2^).

### Treatment of PA and RAS

Adrenalectomy was conducted by experienced urologists in patients with unilateral PA who were willing to undergo surgery. Adrenal lesions were categorized as aldosterone-producing adenoma or unilateral adrenocortical hyperplasia (35). Aldosterone-producing adenomas were diagnosed based on the “Five Corners” criteria recommended by Seccia et al. (35). Unilateral adrenocortical hyperplasia included nodular hyperplasia, which was characterized by multiple nodules in the adrenal gland with cells similar to those in normal tissue, and diffuse hyperplasia, which was characterized by enlarged cytoplasmic volumes with increased lipid content (36). The drug treatment algorithm for patients with bilateral PA or those who were unsuitable for surgery was consistent with that in our previous report (37). Revascularization was recommended if the degree of RAS was > 70% and the patients were able and willing to undergo an interventional procedure (38).

### Data Analyses

Data were analyzed using IBM SPSS Statistics software, version 24.0 (IBM Corp.; Armonk, NY, USA). All continuous variables satisfying the homogeneity of variance assumptions, as confirmed by the Levene test (two-sided *P* > 0.05), are expressed as means ± standard deviation. Paired *t*-tests were used to evaluate changes in continuous variables in the same patient. Categorical variables are expressed as numbers and percentages. Differences between groups were evaluated using the chi-square test or Fisher's exact test for categorical variables and the independent Student's *t*-test for continuous variables.

## Results

### Characteristics of Patients

**Figure 1:**
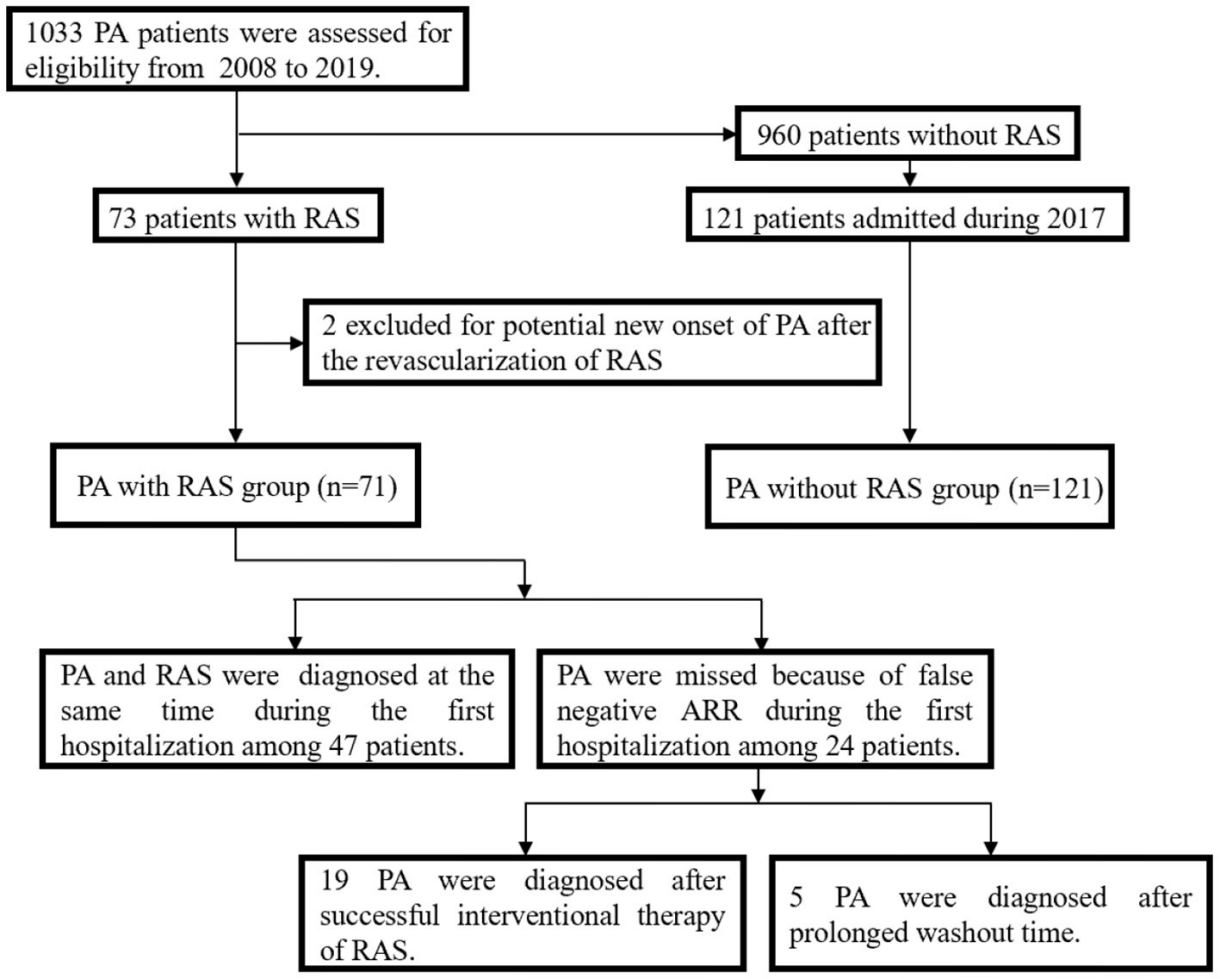
Flow chart depicting the selection of the two groups, and the diagnostic process for patients in the primary aldosteronism (PA) with renal artery stenosis (RAS) group.

In total, 1033 patients were diagnosed with PA from January, 2008 to December, 2019, and 71 of these were also diagnosed with RAS, including 33 with severe RAS and 38 with moderate RAS. These 71 patients were enrolled in the PA with RAS group, and 121 patients with PA only were included in the PA without RAS group (Figure 1). The baseline characteristics of the study participants are summarized in Table 1. The PA with RAS group and PA without RAS group did not significantly differ in age, body mass index, or mean disease duration. The proportion of male patients and history of smoking were significantly higher in the PA with RAS group compared with that in the PA without RAS group. Patients in both groups manifested HTN at admission; however, the mean levels of systolic BP (SBP) and diastolic BP (DBP) and the percentage of patients with RH were significantly higher in the PA with RAS group. Although the absolute blood lipid and glucose levels appeared higher in the PA with RAS group than in the PA without RAS group, a statistically significant difference was only found in total cholesterol. Serum creatinine levels were significantly higher, and the estimated glomerular filtration rate was significantly lower in the PA with RAS group than in the PA without RAS group.

**Table 1:** Medical records of the subjects in the PA with RAS group and PA without RAS group at baseline.

**Characteristics**	**PA with RAS (*****n*** **=** **71)**			**PA without RAS (*n* = 121)**	***P*-value^a^**	***P*-value^b^**	***P*-value^c^**	***P*-value^d^**
	**All (*n* = 71)**	**Severe RAS (*n* = 33)**	**Moderate RAS (*n* = 38)**					
Age, years	53.11 ± 13.40	52.06 ± 12.33	54.03 ± 14.37	50.61 ± 10.59	0.155	0.502	0.115	0.541
Male (%)	54 (76.1%)	24 (72.7%)	30 (78.9%)	66 (54.5%)	**0.003**	0.060	**0.007**	0.540
Disease duration, years	13.10 ± 9.06	14.05 ± 8.53	12.29 ± 9.54	13.88 ± 11.89	0.634	0.942	0.451	0.419
Body mass index, Kg/m^2^	25.68 ± 3.48	25.89 ± 3.23	25.49 ± 3.73	25.54 ± 3.39	0.785	0.588	0.940	0.628
Systolic blood pressure, mmHg	163.55 ± 21.31	171.82 ± 18.24	156.37 ± 21.38	154.11 ± 18.96	**0.002**	**<0.001**	0.537	**0.002**
Diastolic blood pressure, mmHg	104.27 ± 16.43	110.76 ± 15.90	98.63 ± 14.90	91.73 ± 12.85	**<0.001**	**<0.001**	**0.006**	**0.001**
Hypertension (%)	71 (100%)	33 (100%)	38 (100%)	121 (100%)	1.000	1.000	1.000	1.000
Resistant hypertension (%)	58 (81.7%)	30 (90.9%)	28 (73.7%)	81 (66.9%)	**0.027**	**0.008**	0.435	0.061
History of smoking (%)	38 (53.5%)	16 (48.5%)	22 (57.9%)	42 (34.7%)	**0.011**	0.148	**0.011**	0.428
History of alcoholism (%)	26 (36.6%)	12 (36.4%)	14 (36.8%)	38 (31.4%)	0.459	0.590	0.533	0.967
Dyslipidemia (%)	37 (52.1%)	16 (48.5%)	21 (55.3%)	54 (44.6%)	0.316	0.693	0.252	0.569
Diabetes mellitus (%)	15 (21.1%)	9 (27.3%)	6 (15.8%)	14 (11.6%)	0.074	**0.016**	0.403	0.237
Hypokalemia (%)	15 (21.1%)	7 (21.2%)	8 (21.1%)	41 (33.9%)	0.060	0.164	0.135	0.987
Serum potassium, mmol/L	3.78 ± 0.52	3.77 ± 0.48	3.79 ± 0.55	3.69 ± 0.48	0.203	0.164	0.270	0.888
Total cholesterol, mmol/L	4.38 ± 0.82	4.50 ± 0.93	4.27 ± 0.71	4.06 ± 0.91	**0.017**	**0.015**	0.194	0.246
Triglyceride, mmol/L	1.88 ± 0.89	2.00 ± 1.09	1.78 ± 0.68	1.69 ± 0.93	0.178	0.107	0.589	0.323
LDLC, mmol/L	2.94 ± 0.63	2.97 ± 0.69	2.92 ± 0.58	2.74 ± 0.70	0.052	0.094	0.155	0.733
Glucose, mmol/L	5.53 ± 1.16	5.91 ± 1.28	5.20 ± 0.95	5.32 ± 1.08	0.200	**0.008**	0.547	**0.011**
Creatinine, μmol/L	79.94 ± 16.12	82.42 ± 16.33	77.78 ± 15.84	67.27 ± 18.32	**<0.001**	**<0.001**	**0.002**	0.230
eGFR, ml/min/1.73 m2	92.83 ± 23.04	89.15 ± 22.01	96.03 ± 23.72	106.99 ± 20.66	**<0.001**	**<0.001**	**0.007**	0.212
Cardiovascular disease (%)	34 (47.9%)	23 (69.7%)	11 (28.9%)	37 (30.6%)	**0.016**	**<0.001**	0.848	**0.001**
Stroke (%)	14 (19.7%)	7 (21.2%)	7 (18.4%)	17 (14.0%)	0.303	0.315	0.511	0.768
Coronary artery disease (%)	18 (22.5%)	13 (39.4%)	5 (13.2%)	18 (14.9%)	0.180	**0.002**	0.793	**0.011**
Congestive heart failure (%)	2 (2.8%)	1 (3.0%)	1 (2.6%)	1 (0.8%)	0.556	0.322	0.384	0.919
Peripheral artery disease (%)	11 (18.3%)	9 (27.3%)	2 (5.3%)	12 (9.9%)	0.095	**0.010**	0.377	**0.011**

*Values are the mean ± SD, or number (%)*.

*eGFR, estimated glomerular filtration rate; LDLC, low density lipoprotein cholesterol; PA, primary aldosteronism; RAS, renal artery stenosis. P-values less than 0.05 are shown in bold in the table*.

a *comparison between the PA with RAS group and PA without RAS group*;

b *comparison between patients with severe RAS in the PA with RAS group and patients in the PA without RAS group*;

c *comparison between patients with moderate RAS in the PA with RAS group and patients in the PA without RAS group*;

d *comparison of patients with severe RAS and moderate RAS in the PA with RAS group*.

In comparison with that of the PA without RAS group, patients in the severe RAS group had significantly higher SBP, DBP, and RH prevalence, whereas the moderate RAS group only showed higher DBP. Significantly higher total cholesterol, glucose levels, and prevalence of diabetes mellitus were observed in the severe RAS group but not in the moderate RAS group when compared with that in the PA only group. Both the severe and moderate RAS groups showed higher serum creatinine and lower estimated glomerular filtration rate than patients in the PA without RAS group.

### Diagnostic Examinations of PA and RAS

The results of the first screening ARRs for the two groups are shown in Table 2. The PACs with both supine and orthostatic postures were not significantly different between the PA with RAS and PA without RAS groups. The mean levels of both the supine and orthostatic DRCs at screening were significantly higher, and the prevalence of low renin status and plasma ARR was significantly lower in the PA with RAS group than in the PA without RAS group. However, such differences were present only in the severe RAS group, but not in the moderate RAS group, in subgroup analysis.

**Table 2:** Results of PA screening tests among subjects in the PA with RAS group and PA without RAS group at baseline.

**Characteristics**	**PA with RAS (*****n*** **=** **71)**			**PA without RAS (*n* = 121)**	***P*-value^a^**	***P*-value^b^**	***P*-value^c^**	***P*-value^d^**
	**All (*n* = 71)**	**Severe RAS (*n* = 33)**	**Moderate RAS (*n* = 38)**					
Supine PAC, ng/dL	23.58 ± 8.43	23.65 ± 9.53	23.52 ± 7.44	22.38 ± 9.13	0.370	0.486	0.490	0.952
Supine DRC, μIU/ml	5.10 ± 6.51	8.32 ± 8.18	2.31 ± 2.29	2.80 ± 2.85	**0.001**	**0.001**	0.315	**<0.001**
Low-renin status (%)	49 (69.0%)	15 (45.5%)	34 (89.5%)	100 (82.6%)	**0.029**	**<0.001**	0.313	**<0.001**
Orthostatic PAC, ng/dL	18.73 ± 10.60	19.62 ± 11.64	17.95 ± 9.70	17.70 ± 9.06	0.478	0.312	0.885	0.511
Orthostatic DRC, μIU/ml	5.67 ± 4.45	7.56 ± 5.21	3.98 ± 2.76	3.71 ± 2.10	**<0.001**	**<0.001**	0.525	**0.001**
Orthostatic ARR, ng dL^−1^/ μIU ml^−1^	4.66 ± 2.81	3.27 ± 2.08	5.90 ± 2.82	5.61 ± 3.38	**0.048**	**<0.001**	0.635	**<0.001**
False negative ARR	24 (33.8)	20 (60.6%)	4 (10.5%)	4 (3.3%)	**<0.001**	**<0.001**	0.177	**<0.001**

*Values are the mean ± SD, or number (%)*.

*ARR, aldosterone/direct renin concentration ratio; DRC, direct renin concentration; PA, primary aldosteronism; PAC, plasma aldosterone concentration; RAS, renal artery stenosis. P-values less than 0.05 are shown in bold in the table*.

a *comparison between the PA with RAS group and PA without RAS group*;

b *comparison between patients with severe RAS in the PA with RAS group and patients in the PA without RAS group*;

c *comparison between patients with moderate RAS in the PA with RAS group and patients in the PA without RAS group*;

d *comparison of patients with severe RAS and moderate RAS in the PA with RAS group*.

In the PA with RAS group, the first PA screening showed 24 false-negative (FNR = 33.8%), 12 (16.9%) probable-positive, and 35 (49.3%) positive ARRs. In the PA without RAS group, the first ARR tests showed 4 false-negative (FNR = 3.3%), 29 (24.0%) probable-positive, and 88 (72.8%) positive ARRs. The FNR for the PA with RAS group was significantly higher than that for the PA without RAS group (*P* < 0.001). However, the FNRs were 60.6% (20/33) and 10.5% (4/38) in the severe and moderate RAS groups, respectively, and the FNR in the severe RAS group was significantly higher than that in the PA with RAS group.

All 71 patients in the PA with RAS group showed adrenal lesions on at least one side. There were 42 (59.1%) patients with unilateral adrenal abnormalities on the left side (32 nodular and 10 thickenings); 20 (28.2%) with unilateral adrenal abnormalities on the right side (16 nodular and 4 thickenings); and 9 (12.7%) with bilateral adrenal abnormalities (3 with bilateral nodules, 2 with bilateral thickenings, 2 with nodules on the right and thickenings on the left, and 2 with nodules on the left and thickenings on the right).

In the PA with RAS group, severe RAS was diagnosed by percutaneous renal arteriography in 25 patients (19 unilateral and 6 bilateral) and non-invasive examination alone in 8 patients. Analyses of radiological findings of lesions and laboratory tests demonstrated that peripheral atherosclerosis, Takayasu arteritis, and fibromuscular dysplasia were separately present in 61 (85.9%), 8 (11.3%), and 2 (2.8%) of the 71 patients, respectively.

### Missed Diagnoses of PA in the PA With RAS Group

The 24 patients with a false-negative ARR in the PA with RAS group were all strongly suspected of having PA during their hospitalization based on the presence of HTN and/or hypokalemia and adrenal lesions. Among these patients, RAS was severe and moderate in 22 and 2 patients, respectively. Nineteen of the patients with severe RAS underwent revascularization treatment; the PA was revealed when they were readmitted for uncontrolled HTN. Another 5 patients showed negative results during the initial screening; however, results of screening tests were thought to be influenced by medications. Therefore, these patients underwent repeated screening after a prolonged washout period and showed positive results soon after RAS removal (Figure 1).

### Treatment and Follow-Up of Patients in the PA With RAS Group

AVS was conducted in 38 patients in the PA with RAS group. Among these patients, 25 cases of unilateral PA were confirmed and 17 of the 25 patients underwent an adrenalectomy. PA in the other 54 patients was treated with mineralocorticoid receptor (MR) antagonist alone or in combination with other types of antihypertensive medications.

**Figure 2:**
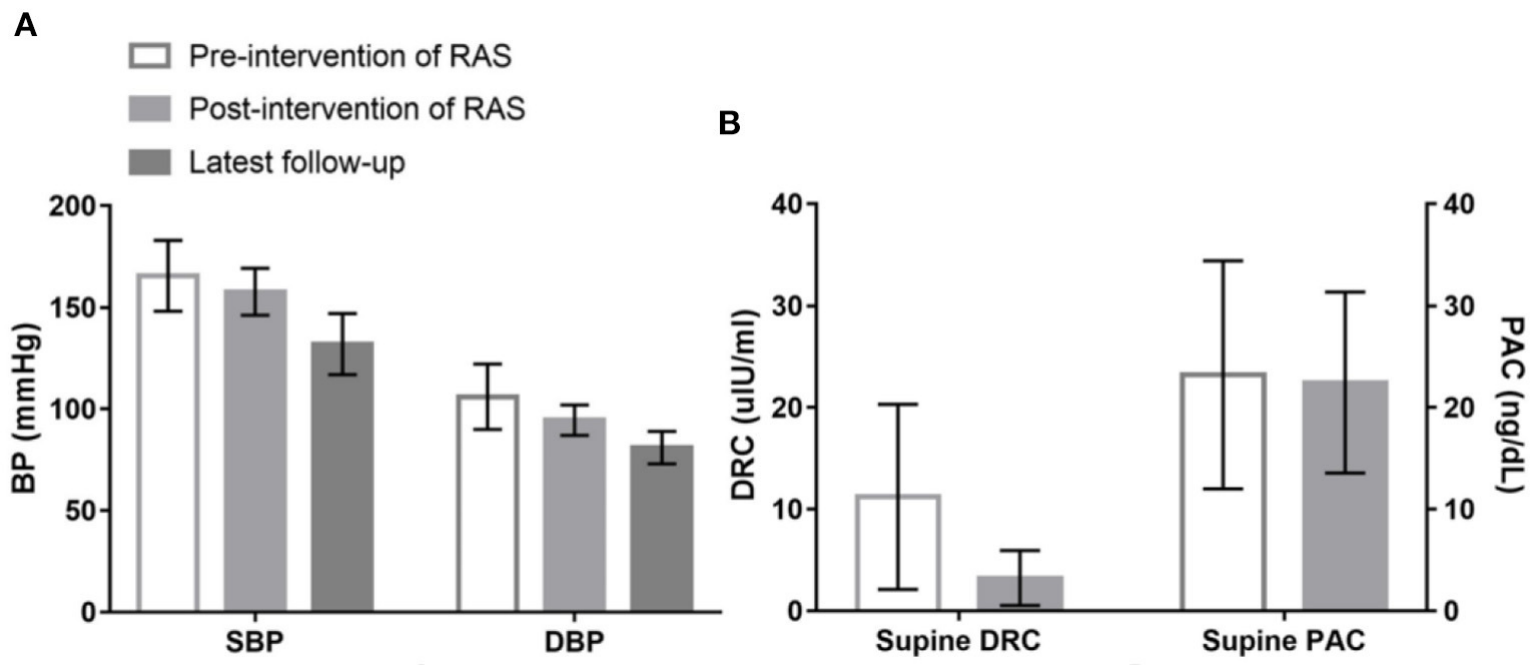
Changes of systolic blood pressure (SBP), diastolic blood pressure (DBP), supine direct renin concentration (DRC) and plasma aldosterone concentration (PAC) among patients underwent revascularization treatment of renal artery stenosis (RAS). **(A)** Shows that compared with the measured values before revascularization treatment for RAS, both SBP (166 ± 17 vs. 158 ± 12 mmHg; *P* = 0.005) and DBP (106 ± 16 vs. 94 ± 8 mmHg; *P* < 0.001) were significantly decreased after revascularization treatment, and SBP and DBP further decreased to 132 ± 15 and 81 ± 8 mmHg, respectively, when those patients received targeted treatment for PA during the latest follow-up. **(B)** Shows that supine DRC lowered from 11.22 ± 9.10 to 3.24 ± 2.69 uIU/ml (*P* < 0.001) compared with the baseline levels before revascularization treatment for RAS; however, no significant difference was observed in PAC (23.22 ± 11.20 vs. 22.45 ± 8.90 ng/dL; *P* = 0.697).

All 25 patients with RAS > 70%, which was confirmed by percutaneous renal arteriography, underwent successful revascularization prior to targeted PA treatments. Compared with the measured values before revascularization for RAS, both SBP and DBP were significantly decreased after revascularization and decreased further when those patients received targeted PA treatment (Figure 2A). Supine DRCs and PACs were re-evaluated in 19 of the 25 revascularization patients when they were re-admitted for a missed diagnosis of PA. The supine DRCs decreased from 11.22 ± 9.10 to 3.24 ± 2.69 μIU/mL (*P* < 0.001) and the prevalence of low renin status increased from 21.1% (4/19) to 78.9 % (15/19) (*P* = 0.001) after revascularization compared with the baseline levels before revascularization treatment for RAS; however, no significant differences were observed for PACs (Figure 2B). After successful revascularization, no significant differences were found for SBP, DBP, DRC, or PAC among patients in the PA with RAS group compared with baseline values from patients in the PA only group; the *P*-values were 0.375, 0.120, 0.552, and 0.977, respectively.

The mean follow-up period was 3.86 ± 1.90 years for the PA with RAS group. At follow-up, the mean SBP (128 ± 12 vs. 164 ± 21 mmHg; *P* < 0.001), DBP (79 ± 7 vs. 104 ± 16 mmHg; *P* = 0.002), and prevalence of RH (2.8 vs. 81.7%; *P* < 0.001) were significantly lower than that which was recorded at baseline.

## Discussion

In this study, we analyzed the clinical features of concurrent PA and RAS. We compared the SBP, DBP, prevalence of RH, DRC, and false-negative rates of PA screening tests between the PA with RAS and PA without RAS groups. The most-notable conclusions from this study are as follows: (I) PA with concurrent severe RAS is a condition that induces RH; (II) PA is easily missed in patients with coexisting RAS; (III) RAS patients with refractory postoperative HTN after successful revascularization of renal artery disease should be evaluated for concurrent PA.

PA with RAS is commonly considered to be a rare condition. However, the prevalence of RAS in PA patients was 6.9% (71/1,033) in this cohort, including 3.2% (33/1,033) with severe RAS. These data may contribute to the establishment of a systemic screening procedure for all patients suspected of having secondary HTN, which includes both an ARR test and CT scan of the renal artery and adrenal gland. We had noticed sporadic cases of PA with severe RAS in clinical practice, among which 50% of PA diagnoses were missed at first admission (39). Therefore, we increased our vigilance and re-evaluated the ARR test for patients who showed uncontrolled HTN after successful revascularization of RAS. As expected, far more cases of PA with RAS were found.

In current evidence, the effect of aldosterone is largely mediated through the MR. The excessive activation of the MR by aldosterone in the kidney results in volume expansion, hypertension, hypokalemia, and metabolic alkalosis (40). In addition to the well-known effects of aldosterone in the kidney, more widespread effects of aldosterone via MR include SNS activation (6, 41) and increased oxidative stress, with inflammation, remodeling, apoptosis, and fibrosis of the cardiovascular tissues (5, 42). These mechanisms are activated by aldosterone through MR in genomic and nongenomic pathways, and the latter through the MR initiate several canonical cells signaling mechanisms depending on the cell type and context (43). In patients with RAS, the hypoperfusion of the juxtaglomerular apparatus stimulates the RAAS and subsequently increases Ang-II, which is the most important stimulator of aldosterone synthesis. Ang-II and aldosterone are pathologically increased in RAS and act synergistically through their respective receptors in the heart, vessels, inflammatory cells, and central nervous system, as well as the kidney, to increase the BP and cause pathological remodeling (16, 44, 45). The mechanisms of HTN and tissue remodeling of PA and RAS are similar, especially those are caused by aldosterone. It is also important to note the difference between PA and RAS is the levels and actions of Ang-II. Meanwhile, Ang-II and aldosterone augment each other actions. Aldosterone upregulates type 1 Ang-II receptors in vascular smooth muscle cells which leads to enhanced vasoconstriction (46). The synergistic effects of aldosterone and Ang II trigger vascular inflammation and fibrosis and contribute to arterial hypertension, which ultimately leads to left ventricular hypertrophy and other adverse cardiac sequelae (47). The results in this research also suggest the pathogenesis of PA combined with RAS. In our study, the mean DRC levels, but not PAC, were significantly different between our two groups, which indicated that over-secretion of renin played an essential role in the induction of higher BP within the PA with RAS group. Only the severe RAS group showed higher SBP, DBP, and RH prevalence, which is consistent with the commonly accepted view that only RAS > 70% is hemodynamically significant. Nevertheless, patients with concurrent PA and moderate RAS also displayed higher DBP than patients with PA only, which is consistent with a previous report (48). These data may indicate that mild-to-moderate RAS also promotes intra-renal renin secretion and SNS activation. Such changes may induce elevated BP but can rarely be observed in a serum sample because renin levels can fluctuate based on other factors, such as salt consumption and intravascular volume (49). Overlap of multiple mechanisms caused by concurrent PA and RAS may induce the high prevalence of RH. Overall, the prevalence of RH decreased from 81.7 to 2.8% (P <0.001) when both PA and RAS were treated with standard methods, which indicates that RH in patients with both PA and RAS is remediable.

PA is characterized by autonomous aldosterone overproduction, which leads to low renin levels. However, it has also been reported that 27.5% of PA patients present with normal or elevated renin levels, which may be because blood volume is not always elevated in PA, for instance, under conditions of salt restriction or the use of diuretics. High renin status is regarded as an indicator of hemodynamic changes in RAS. However, low renin renovascular HTN has also been reported previously, which may be attributed to renal ischemia, renal failure, or underestimation of renin levels because of technical problems and/or drug interference (50, 51). The RAAS status is inevitably far harder to interpret in patients with PA and RAS. In this study, the PA with severe RAS group had a significantly higher mean DRC and a decreased percentage of patients with low renin than the PA without RAS group; at admission, however, no difference was found between PA patients with moderate RAS and those without RAS. Based on these findings, we conclude that severe RAS diminishes the PA-mediated reduction in DRC, and this effect may be reversed by successful intervention for RAS. Neither the supine nor orthostatic PAC values were significantly different between the groups, which led to significantly decreased ARRs based on low levels of DRC and a higher FNR in the patients with concurrent PA and severe RAS. The autonomous secretion of aldosterone by definition is independent of renin and Ang-II in PA patients. However, the secretion of aldosterone is regulated by angiotensin-converting enzymes, body fluid volume, and other factors such as extracellular potassium concentration (52).

A comparison of baseline data indicated that the proportion of male patients was statistically different between the two groups. This result is consistent with the prevalence of RAS, which is associated with the male sex (53), and demonstrates that concurrent PA and RAS is more likely to occur in male patients. However, in premenopausal, ovulating women, plasma aldosterone levels rise rapidly in the luteal phase. Because renin levels are lower, the ARR is higher than in men for all phases of the cycle, but especially during the luteal phase in which aldosterone increases to a greater extent than renin (18). As a result, false positives can occur during the luteal phase in women (1). However, the subjects in our study were patients with a precise diagnosis of PA, and we did not include patients with false-positive ARRs. Therefore, this situation does not affect our results. Because the ARR is a biochemical case-detection test, it inevitably yields false-positive and false-negative results for other reasons (20). We addressed the potential factors that reportedly affect DRC and PAC levels before screening and confirmatory tests by ceasing related medicines for a washout period, treating hypokalemia, and taking normal dietary sodium (18, 54). However, the results of PA diagnostic tests may be influenced by coexisting RAS. Although renal artery disease is widely considered to affect the results of PA screening tests (18, 54), data from patients with both PA and RAS are limited.

The relationship between PA and RAS remains unclear. It is difficult to determine in a retrospective study whether RAS is secondary to PA or PA is secondary to RAS. However, the association between PA and atherosclerotic peripheral artery stenosis has been confirmed in multiple studies (55–57). Atherosclerosis is the most common etiology of RAS and accounts for over two-thirds of RAS patients followed by fibromuscular dysplasia, Takayasu arteritis, and other rare conditions (58). In our cohort, 85.9% (61/71) of the PA with RAS patients were diagnosed with atherosclerotic RAS on the basis of radiologic features; the rare etiologies were Takayasu arteritis and fibromuscular dysplasia. PA is a common factor that induces atherosclerotic RAS (59). Current evidence indicates that overproduction of aldosterone leads to inflammation and oxidative stress, vascular endothelial cell dysfunction, and vascular remodeling (5), all of which are pathological mechanisms that can induce peripheral atherosclerosis (60, 61). Accumulation of various collagen fibers, inflammatory factors, and growth factors in vascular smooth muscle cells was found to be related to hyperaldosteronism in animal experiments (31, 62, 63). However, little evidence has been found regarding the pathophysiological effects of RAS on PA. A hypothesis referred to as “tertiary” aldosteronism proposes that chronic renin stimulation of the adrenal glands caused by RAS-related kidney ischemia may result in autonomous aldosterone secretion (64). However, this hypothesis remains unproven.

This study had several limitations. First, bias is inevitable based on the retrospective and single-center study design. We did acknowledge that approximately half of the patients with both PA and RAS underwent lateralization procedures and only 68% of patients with a unilateral aldosterone producing adenoma had unilateral adrenalectomy. The appropriate definitive treatment was not implemented, and the fact that the remaining patients were treated with MR antagonists may have affected the clinical and biochemical parameters that were assessed. A prospective multicenter study will provide stronger clinical evidence. Second, for the 19 patients whose first PA screening tests were negative but who were diagnosed with PA after revascularization surgery for RAS, we cannot definitively reject the possibility that PA may have originated after surgery. However, all 19 patients presented with adrenal lesions and persistent uncontrolled HTN after surgery, and those showing recurrent or aggravated HTN after surgery were excluded, which maximized the detection of concurrent PA and RAS at first admission. Third, the evaluation method for renin profiling was different before and after 2016, and PRA (ng/mL/h) was converted to DRC (mIU/L) using the recommended conversion factor of 8.2 during the statistical analysis, which may have introduced errors. Although there is evidence showing a weaker correlation between DRC and PRA values <1 ng/mL/h (65), conversion of these data was only conducted for statistical analysis and was not involved in the diagnostic process. We considered the conversion to be necessary because patients with concurrent PA and RAS are extremely rare and not using the conversion would have produced a smaller cohort, possibly introducing more bias.

## Conclusion

In summary, patients who had PA with severe RAS showed a higher prevalence of RH. However, PA is easy to miss in PA patients with coexisting severe RAS because of the high FNR on screening tests. Patients with refractory postoperative HTN after successful revascularization for renal artery disease should be evaluated for coexisting PA.

## Data Availability Statement

The original contributions presented in the study are included in the article, further inquiries can be directed to the corresponding author/s.

## Ethics Statement

The studies involving human participants were reviewed and approved by Ethics Committee of Fuwai Hospital. The requirement for informed consent was waived because of the retrospective nature of the study.

## Author Contributions

LZ, JHX, XPD, MX, and XLZ contributed to conception and design of the study. LZ and JHX organized the database. LZ, JHX, YZ, XQD, and FL performed the statistical analysis. LZ and JHX wrote the first draft of the manuscript. YZ, FL, and JHX wrote sections of the manuscript.

## Funding

This work was supported by the Non-profit Central Research Institute Fund of Chinese Academy of Medical Sciences (2019XK320057), CAMS Innovation Fund for Medical Sciences (2016-I2M-1-002), the National Key Research and Development Program of China (2016YFC1300100), and Tianjin Binhai New Area Science and Technology Project (Grant No. BHXQKJXM-SF-2018-11). The funders had no role in study design, data collection and analysis, decision to publish, or preparation of the manuscript.

## Conflict of Interest

The authors declare that the research was conducted in the absence of any commercial or financial relationships that could be construed as a potential conflict of interest.

## Publisher's Note

All claims expressed in this article are solely those of the authors and do not necessarily represent those of their affiliated organizations, or those of the publisher, the editors and the reviewers. Any product that may be evaluated in this article, or claim that may be made by its manufacturer, is not guaranteed or endorsed by the publisher.
